# Emerging advances in nanobiomaterials-assisted chimeric antigen receptor (CAR)-macrophages for tumor immunotherapy

**DOI:** 10.3389/fbioe.2023.1211687

**Published:** 2023-06-14

**Authors:** Yanan Zhang, Jingxing Yang, Tinghao Zhang, Hongchen Gu

**Affiliations:** ^1^ Nano Biomedical Research Center, School of Biomedical Engineering, Med-X Research Institute, Shanghai Jiao Tong University, Shanghai, China; ^2^ Shanghai Datong High School, Shanghai, China

**Keywords:** cancer immunotherapy, nanobiomaterials, chimeric antigen receptor, tumor-associated macrophages (TAMs), genetic engineering

## Abstract

Adoptive cell immunotherapy, especially chimeric antigen receptor (CAR)-T-cells therapy, has made great progress in the clinical treatment of hematological malignancies. However, restricted by the complex tumor microenvironment, the potential efficiency of T-cell infiltration and activated immune cells are limited, thus failure prevented the progression of the solid tumor. Alternatively, tumor-associated macrophages (TAMs), one sustentacular and heterogeneous cellular population within the tumor microenvironment, are regarded as potential therapeutic targets. Recently, CARs have shown tremendous promise in treating malignancies by equipping macrophages. This novel therapeutic strategy circumvents the tumor microenvironment’s limitations and provides a safer therapeutic approach. Meanwhile, nanobiomaterials as gene delivery carriers not only substantially reduce the treatment cost of this novel therapeutic strategy, but also set the foundation for *in vivo* CAR-M therapy. Here, we highlight the major strategies prepared for CAR-M, emphasizing the challenges and opportunities of these approaches. First, the common therapeutic strategies for macrophages are summarized in clinical and preclinical trials. Namely, TAM-targeted therapeutic strategies: 1) Inhibit monocyte or macrophage recruitment into tumors, 2) deplete TAMs, and 3) reprogramme TAMs to antitumor M1 phenotype. Second, the current development and progress of CAR-M therapy are reviewed, including the researchers’ attempts in CAR structure design, cell origin, and gene delivery vectors, especially nanobiomaterials as an alternative to viral vectors, as well as some challenges faced by current CAR-M therapy are also summarized and discussed. Finally, the field of genetically engineered macrophages integration with nanotechnology for the future in oncology has been prospected.

## 1 Introduction

Immunotherapy has made revolutionary progress in cancer treatments and has become mainstream in clinical trials. Specifically, the adoptive transfer of immune cells immunotherapy, for instance, chimeric antigen receptor (CAR)-T cells has brought a new dawn for patients who suffered hematological malignancies. Different from other therapeutics, CAR-T activates in advance to recognize the specific tumor cells *in vivo*, avoiding the complex immune activation pathway, which makes this approach more efficient (Chen and Mellman, 2013). Hence, genetically modified T-cells expressing CARs present one of the most prominent ways of cellular immunotherapy, which activate immune cells to exert anti-tumor effects through the specific recognition of tumor-specific antigens by antibodies as well as the downstream signaling of T-cells (June et al., 2018). Due to their prominent therapeutic effects in many hematological malignancies, the US Food and Drug Administration (FDA) has approved six kinds of drugs for B-cell maturation antigen (BCMA) hitherto (Schuster et al., 2017; Abramson et al., 2020; Raychaudhuri et al., 2020; Khurana et al., 2021; Si Lim et al., 2021; Ying et al., 2021). In stark contrast, in patients with solid tumors, CAR-T therapy has not been shown to be effective (Narayan et al., 2022; Patel et al., 2022). Plenty of research has explored the mechanism, in which tumor microenvironment (TME) immunosuppressive properties and poor infiltration of T lymphocytes into the tumor joint harmed the effectiveness of treatment for solid tumors (Kumar Vodnala and Restifo, 2019; Elia and Haigis, 2021; Huang et al., 2021; Kim et al., 2022). Additionally, the limited durability of CAR-T, inefficient response rate caused by antigen escape, cytokine release syndrome (CRS), and neurotoxicity, as well as tedious and complex preparation processes, limit their clinical applications in a variety of malignant tumors (Hou et al., 2021; Marofi et al., 2021; The Lancet Oncology, 2021; Kyte, 2022). To overcome the diminished efficacy against solid tumors, introduction CARs into other innate immune cells provides revolutionary innovation in cancer immunotherapy. Among them, macrophages provide an ideal alternative and exhibited promising potency for CAR-modified strategy against solid tumors.

**FIGURE 1:**
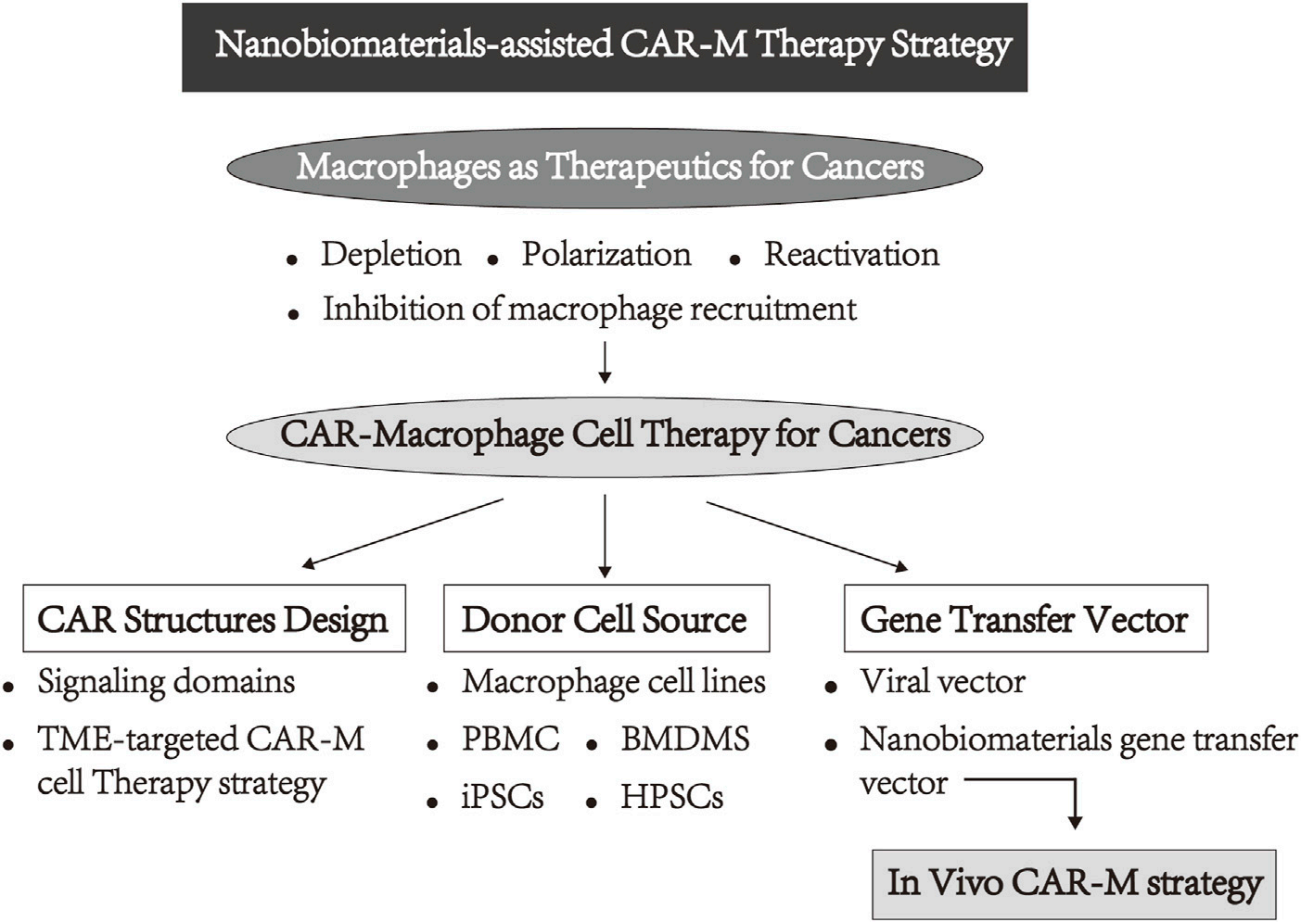
Schematic view of the layout of this review.

Macrophages are highly plasticity and polarization, and they play a double-edged sword in cancer therapy (Locati et al., 2020). First, macrophages can infiltrate tumors more efficiently than other immune cells, resulting in their highest proportion (more than 50%) in TME (Wagner et al., 2019). In addition, the growing evidence indicates that the maintenance of the treatment-resistant microenvironment to support the development and metastasis of tumors are closely related to macrophages, particularly TAM, in many malignancies tumor-bearing mice models and clinical trials, such as breast cancer, lung cancer, ovarian cancer, colorectal cancer, glioblastoma, and other cancers (Wenes et al., 2016; Halbrook et al., 2019; Lin et al., 2019; Singhal et al., 2019; Yin et al., 2019; Buonfiglioli and Hambardzumyan, 2021; Lu et al., 2021; Huang et al., 2022). More importantly, as part of the innate immune system, macrophages play a central role as effectors and regulators, and the activated macrophages present phagocytosis, cytotoxicity, as well as the function of secreting pro-inflammatory factors and presenting antigen to T-cells, which have great potential in activating anti-tumor innate immune and adaptive immune responses via immune cascade response (DeNardo and Ruffell, 2019; Mantovani et al., 2022). Therefore, these above-mentioned characteristics and inherent advantages of macrophages seem to be better suited for genetic modification to circumvent the barriers faced by CAR-T. Recently, the CAR-M strategy has also attracted extensive attention both in the laboratory and in the industry, and research reports on the feasibility of the CAR-M technology, safety, and therapeutic effect were preliminarily explored. In these existing explorations, researchers have placed significant emphasis on the design of CAR structure, the selection of donor cell sources, and the vector delivery of CAR genes into macrophages. Simultaneously, the CAR-M treatment strategy has evolved from *in vitro* modification of macrophages to the direct *in vivo* administration of gene reagents for *in situ* macrophage modification. In the development of CAR-M therapy, the gene delivery vector has emerged as a critical concern that restricts the effectiveness and applicability of this strategy. Due to outstanding advantages in transduction efficiency, viral vectors have become the most widely used in the field of cell therapy (Bin Umair et al., 2022). Various viral vectors, including adenoviral vectors and lentiviral vectors, have been applied to CAR-M therapy. However, the high cost and safety problems of viral vectors interfered with the development of CAR-M therapy. Differently, nonviral vectors represented by nanobiomaterials become an attractive alternative due to the advantages in the simplicity of the production process, ease of large-scale production, and lack of specific immune response (Ramamoorth and Narvekar, 2015; Zu and Gao, 2021). At present, with the above unique characteristics and advantages, a variety of nanobiomaterials including LNP formulations, cationic polymers, and biocompatible hydrogels have been employed in the development of CAR-M therapy. These materials serve as alternatives to viral vectors for delivering CAR genes and exhibit great potential in the context of *in vivo* CAR-M therapy strategies (Figure 1). In this review, we elaborate the CAR-M therapy’s research progress and clinical attempts and discuss ongoing innovations in this approach to enhance clinical efficacy in solid tumors, including the CAR structures design, the cellular origin of macrophages, and the use of novel nanometer biomaterials, as well as the challenges and opportunities of CAR-M-based immune cell therapy that have emerged in clinical and preclinical studies.

## 2 Macrophages as therapeutics

**FIGURE 2:**
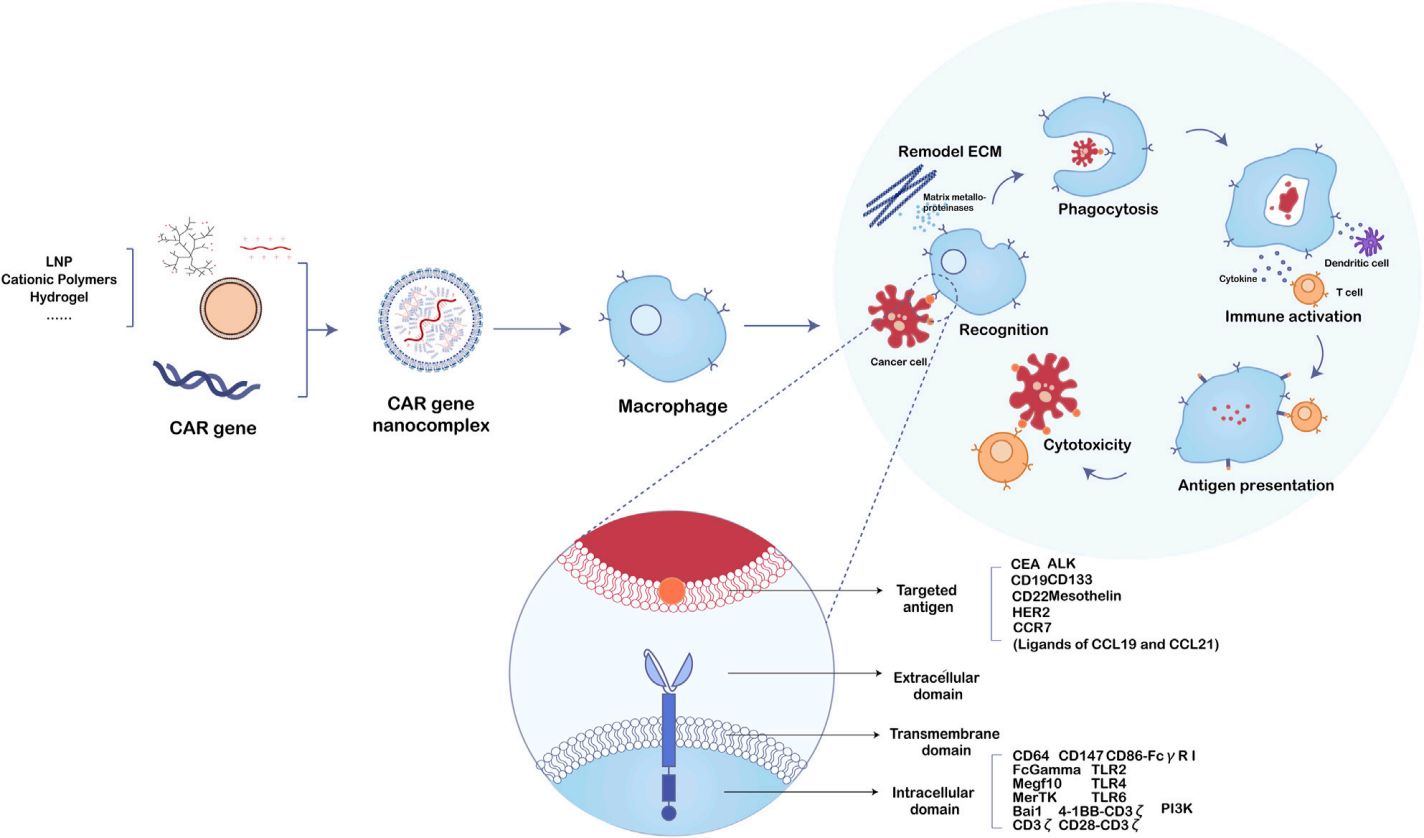
The function mechanism of nanobiomaterials-assisted chimeric antigen receptor (CAR)-macrophages for tumor immunotherapy.

Macrophages are a diverse and intricate group of cells infiltration the tumor microenvironment (Wagner et al., 2019; Christofides et al., 2022). Based on the classical paradigms of M1 and M2 phenotypes with pro-inflammatory and anti-inflammatory properties, the mainstream anti-tumor therapy for TAM mainly focuses on the strategies for depletion (Mazzieri et al., 2011; Germano et al., 2013), remodeling TAM including polarization or reactivation (Hoves et al., 2018; Eisinger et al., 2020; Yang et al., 2020; Anfray et al., 2021; Sun et al., 2021; Nie et al., 2022), and inhibition of monocyte or macrophage recruitment into tumors (Qian et al., 2011; Mantovani et al., 2017; Cassetta and Pollard, 2018). Recently, a large number of clinically developed drugs mainly focus on the targets CSF-1R, CD47, CD40, and chemokines (Beatty et al., 2011; Yanagita et al., 2017; Sikic et al., 2019; Sylvestre et al., 2020). However, restricted by the high plasticity of macrophages and the lack of specificity of tumor-associated antigens, these single therapies for solid tumors have only achieved limited therapeutic effects with substantial off-target toxicity. Hence, various combination therapy strategies based on macrophages have been extensively developed. Wang et al. designed a sonoimmunity-engineered nanoplatform to reverse the physical microenvironment of the tumor by producing a substantial amount of reactive oxygen species (ROS) through the sonodynamic processes (Wang et al., 2022a). The substantial accumulation of ROS within tumors induces the polarization of TAMs towards the pro-inflammatory M1 subtype, thereby amplifying the therapeutic efficacy of immune checkpoint blockade and CAR-T therapy against solid tumors with metastasis. A similar strategy was reported by Powell Jr.’s Group (Rodriguez-Garcia et al., 2021), and they proved the selective elimination of FRβ+ TAMs by CAR-T cells enriched pro-inflammatory monocytes and stimulated tumor-specific CD8^+^ T-cells to infiltrate in the TME, eventually syngeneic tumor-bearing mice have delayed tumor progression and prolonged survival rate. These paradigms that change one point yet benefit the whole provide a rationale for using CAR-M in patients with solid cancers. Different from the traditional TAM-targeted treatments, using chimeric antigen receptor expression on the macrophage surface is a whole new attempt. This method hypothesized that macrophages expressing the chimeric antigen receptor could simultaneously boost phagocytosis, change M2-to-M1 phenotypes, and activate adaptive immunity (Figure 2) (Morrissey et al., 2018; Klichinsky et al., 2020; Kang et al., 2021; Niu et al., 2021). Since Morrissey et al. verified the phagocytosis of macrophages affected by various CAR structures (Morrissey et al., 2018), more and more studies confirmed this innovative concept. Interestingly, CAR-T which acts directly on macrophages rather than cancer cells also achieved prominent therapeutic effects on breast cancer and colorectal cancer models simultaneously. For instance, Chen et al. delivered the CD47 blocker SIRPα-Fc by CAR-T cells enhancement antitumor efficacy (Chen et al., 2022b), while Achkova et al. (2022) designed CAR-T-cells that contain M-CSF and IL-34 to mediate selective remodeling of macrophage cells that express enforced or endogenous M-CSFR. Also the synchronous secretion of cytokines including IL-2 and interferon (IFN)-γ suppressed tumor cell progression. Comprehensively, CAR-M technology might represent a novel therapeutic approach for manipulating the TAM to become an M1 phenotype, enhance the activity of phagocytosis or antigen presentation to further induce the immune response, and sensitize other common treatments, which hold potential against solid tumors.

## 3 CAR-macrophage cell therapy for cancers

### 3.1 Milestones of CAR-Macrophage in cancer therapy

**FIGURE 3:**
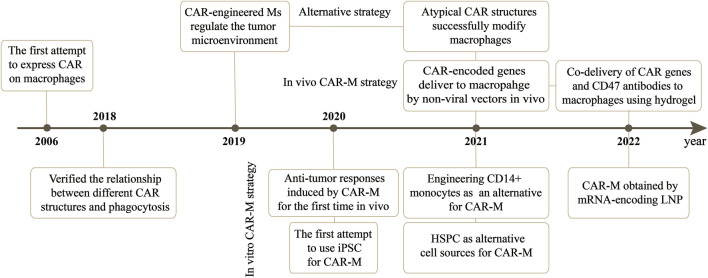
The development of CAR-M therapy and the gene delivery vectors applied in this strategy.

**FIGURE 4:**
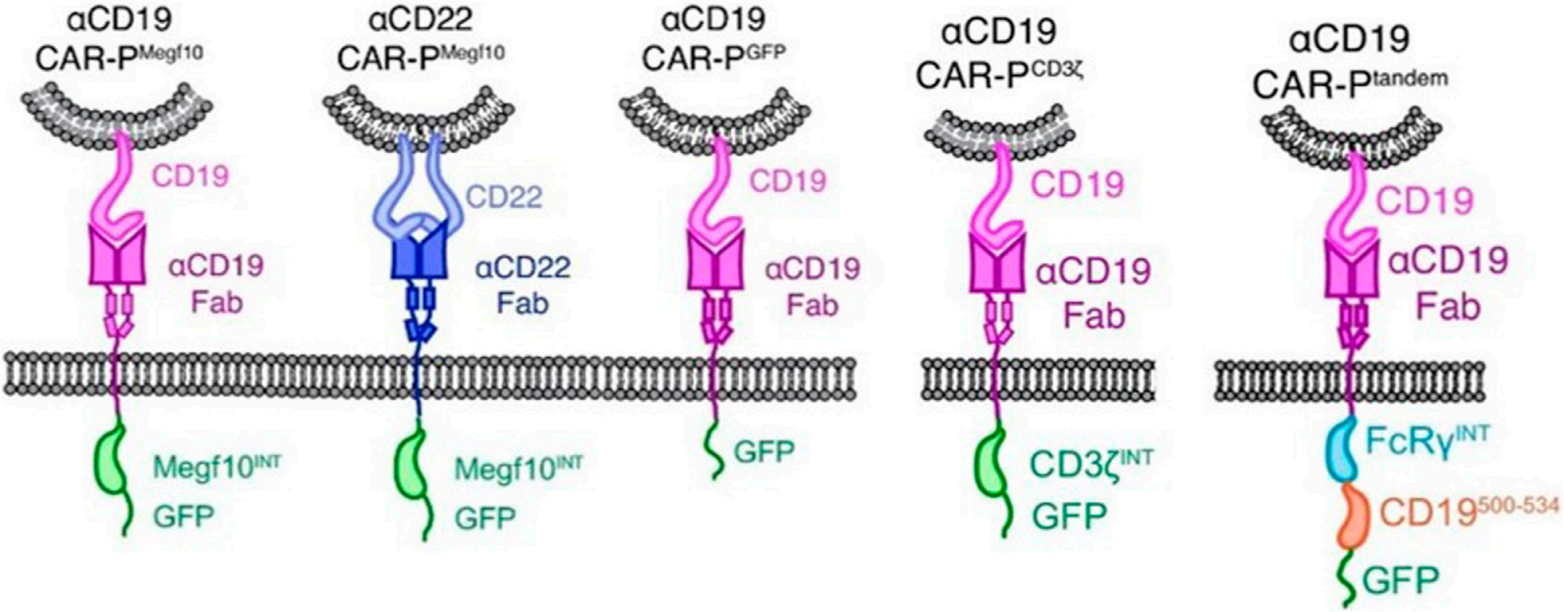
Schematics show the structure of CAR-P constructs (Morrissey et al., 2018). Copyright^©^ 2018, Morrissey et al.

CAR-M therapy is an iteration of CAR-T or CAR-NK cell therapy that has undergone several updates to date (Figure 3). The earliest attempts to express CARs on macrophages came from Biglari et al. (2006). They created a chimeric CD64 molecule containing an scFv that targets the human carcinoembryonic antigen (CEA) and transmembrane and the cytoplasmic domain of human CD64. After the corresponding expression gene was delivered to human monocytes using an adenoviral vector, such monocytes expressing chimeric CD64 receptors could induce the secretion of antigen-specific cytokine when monocytes recognized CEA proteins or CEA-expressing tumor cells, and eventually significantly reduce the growth rate of tumors in the xenotransplantation model. Nevertheless, except for the monocytes, this attempt is not an integral macrophage expressing a CAR molecules structure that lacks intracellular stimulation signal domain. Subsequently, Morrissey et al. (2018) triggered the real wave of CAR-T technology application to macrophage transduction. A novel category of synthetic receptors, functionalized as Chimeric Antigen Receptors for Phagocytosis (CAR-Ps), was designed, and successful engineering of macrophages was achieved. Within the CAR-P molecules, the extracellular single-chain antibody variable fragment (scFv) exhibits the ability to recognize the B cell antigens CD19 or CD22. Additionally, through screening a library of known murine phagocytic receptors, intracellular signaling structures (Megf10, FcRɣ, and CD3ζ) were identified, which effectively enhance the phagocytic function of macrophages (Figure 4). This work demonstrated that CAR-M could identify and ingest targets through specific antibody-mediated interactions. Further exploring the mechanism, they confirmed the CAR method can be transferred to biological processes other than T-cell activation and that the expression of engineered receptors in macrophages was sufficient to promote specific phagocytosis and the elimination of cancer cells. Since then, researchers have developed a range of CAR structures specifically designed for macrophage modification, and additional signaling domains have been found to be applicable to CAR-M treatment strategies. As summarized by Wang et al. (2022b), through iterative refinement of the CAR structure, the phagocytic capacity of CAR-M has been strengthened, along with improvements in tumor-associated antigen presentation and promotion of T-cell activation functions. Recently, Klichinsky from the University of Pennsylvania demonstrated that an effective antitumor response could be achieved with CAR-M *in vivo* for the first time (Klichinsky et al., 2020). The authors utilized a chimeric adenoviral vector (Ad5F35) to efficiently transduce CAR molecules that could specifically target the HER2 or mesothelin antigen into human macrophages, resulting in efficient and sustained CAR expression in macrophages. In the subsequent anti-tumor experiment in five different xenograft models, these modified macrophages generated against solid tumor response and significant reduction in tumor burden and the prolongation of mice survival both in intravenous and subcutaneous administration. Interestingly, this CAR transduction successfully induced the transformation of macrophages to a pro-inflammatory phenotype, activated the lymphocyte T-cells into tumors, and reversed the tumor-suppressive microenvironment. Inspired by these outstanding results, the authors from the same group sponsored a phase I trial (NCT04660929) to assess the therapeutic efficiency of CAR-M in patients with metastatic HER2-overexpressing tumors for the first time. In 2022, they disclosed the safety of CAR-M in clinical patients, but its effectiveness including overall response rate and progression-free survival is still being tested.

### 3.2 Challenges and opportunities of CAR-Macrophage cell therapy for solid tumors

#### 3.2.1 TME-targeted CAR-M cell therapy strategy

**FIGURE 5:**
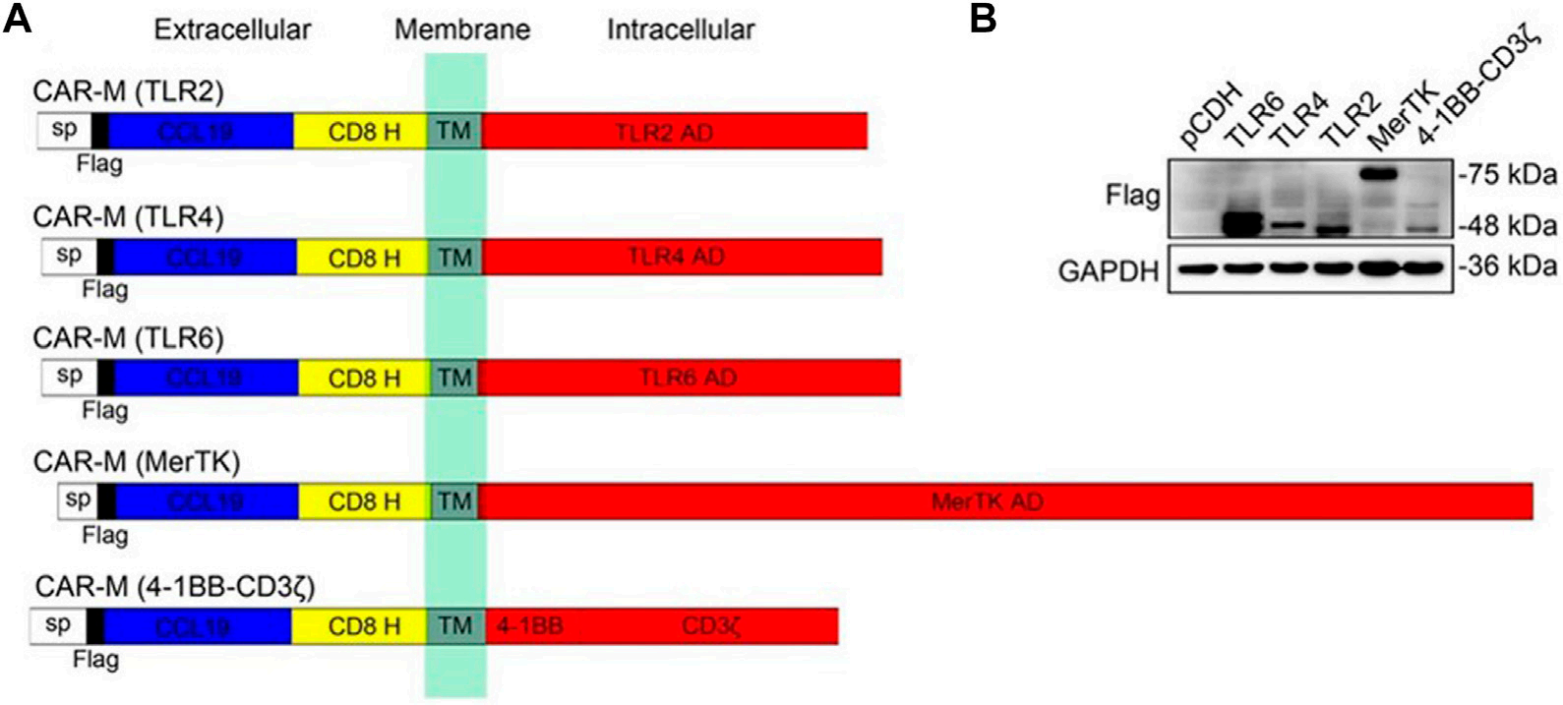
Construction and identification of CAR-Ms that target CCR7. **(A)** Schematics show the structure of five different types of CAR-M. **(B)** Immunoblotting results in RAW264.7 CAR-M cells (Niu et al., 2021). Copyright^©^ 2021, Wiley.

For solid tumors, the dense tumor physical microenvironment formed by the extracellular matrix (ECM) is considered to be one of the main barriers that hinder CAR-cell infiltration, thus hindering therapeutic efficacy. To overcome this challenge, Zhang et al. designed a CAR structure consisting of a single-chain fragment variable (ScFv) fragment targeting human HER2, IgG1 hinges, and mouse CD147 transmembrane and intracellular regions (CAR-147) (Zhang et al., 2019). The macrophage-expressing matrix metalloproteinases were activated by intracellular CD147 molecules and subsequently degraded the tumor ECM to overcome physical barriers. To enhance the effectiveness of CAR-M in the treatment of solid tumors, the researchers further engineered the CAR molecule to stimulate the tumor therapeutic potential of CAR-M cells, such as using innovative extracellular receptors to modify the CAR molecular structure or adding intracellular domains and co-stimulating domains to improve the role of macrophages in presenting tumor-associated antigens and activating T-cell. More recently, Niu et al. (2021) designed to utilize the native ligand CCL19 of the CCR7 as an antigen recognition domain for the receptor, rather than scFv to secrete the classical T-attractive chemokines for recruiting the lymphocytes. For intracellular domains, they evaluated multiple types of common activation domains (Figure 5). Experiments confirmed that the cytoplasmic domain of the Mer receptor tyrosine kinase (MerTK) can also trigger cytotoxicity on tumor cells through CAR-M, and *in vivo*, the CAR-M that targets CCR7 prevents tumor metastasis and delays LDhiCCR7hi immunosuppressive cells from migrating from tumor tissue to distal organs, which prolongs survival and inhibits tumor growth.

#### 3.2.2 Donor cell source

**FIGURE 6:**
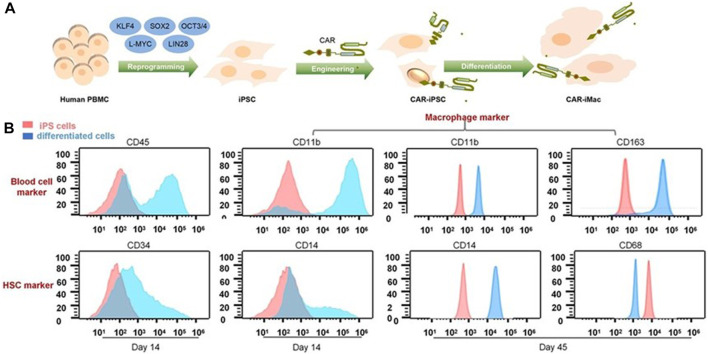
CAR-expressing iPSCs can differentiate into CAR-macrophage cells. **(A)** Overview of the process of deriving CAR-iMacs from CAR-iPSCs. **(B)** Flow cytometry analysis of different stages of differentiation in iPSC-derived cells (Zhang et al., 2020). Copyright^©^ 2020, the Author(s).

**FIGURE 7:**
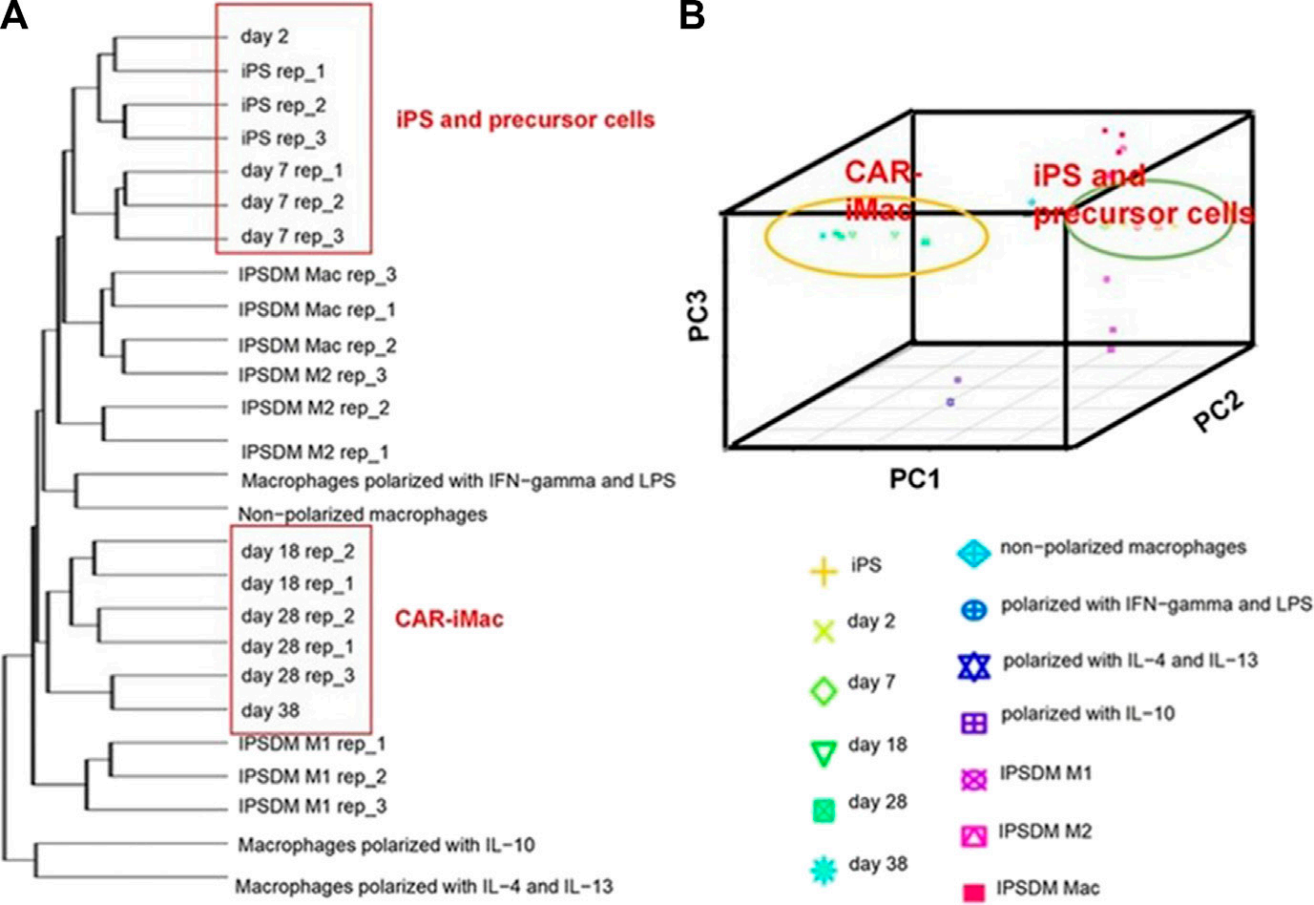
CAR-expressing iPSCs can differentiate into CAR-macrophage cells. **(A)** Hierarchical clustering of transcriptomes of CAR-iPSCs in different states. **(B)** Principal component analysis (PCA) of the same samples as in **(A)** (Zhang et al., 2020). Copyright^©^ 2020, the Author(s).

As previously mentioned, although Klichinsky proved the effectiveness of CAR-M *in vivo*, the production of genetically modified cells is still *in vitro*. It is worth pointing out that the expansion of macrophages is difficult to achieve, which is a huge obstacle to the large-scale application and development of CAR-M technology. Klichinsky et al. averted or solved this challenge to some extent by extracting CD14 monocytes from the patient’s peripheral blood for genetic engineering and then inducing differentiation into macrophages, however, a potential disadvantage of autologous monocyte production of CAR-M is that the number and function of circulating monocytes in cancer patients may be affected by previous treatments, thereby hindering adequate monocyte isolation. Gabitova, also from Carisma Therapeutics, sought to assess whether it was feasible to directly engineer CD14^+^ monocytes to express CAR molecule in order to simplify the manufacturing process (Gabitova et al., 2021), increased cell yield, and potentially improved tumor infiltration. With the engineered adenovirus vector (Ad5f35), CAR monocytes were designed to target HER2-overexpressing tumors. CAR-monocytes could efficiently differentiate into macrophages expressing CAR molecule after 3–5 days, secrete pro-inflammatory cytokines, and specifically kill HER2 overexpression target cells. More notably, they built a closed system that could ultra-quickly engineer monocytes to optimize CAR-M’s manufacturing process. This process enabled the production of CAR monocyte products to be completed within 1 day, significantly reducing the treatment cost and treatment time. The establishment of this platform was of epoch-making significance for CAR-M to move towards automation. In another study, Zhang et al. (2020) provided an extra strategy to solve the expansion problem by extracting induced pluripotent stem cells (iPSCs) from peripheral blood monocytes of healthy donors (Figure 6). First, they used lentiviral transduction to introduce CAR structure into iPSCs and then induced the differentiation of iPSCs into the bone marrow cell lineage by a bone marrow/macrophage differentiation protocol (Figure 7), finally got a sufficient number of CAR-iMacs. This technology for the first time provided iPSC-derived engineered CAR macrophages from unlimited sources, breaking through the shortcomings of the long preparation cycle and small-scale production in the macrophage therapy strategy, compared to the common autologous peripheral blood sources. However, there are still some unsolved problems in the application field of iPSC, such as tumorigenic risk, high immunogenicity, and low induction efficiency. To overcome these challenges, Paasch et al. (2022) proposed a novel strategy by introducing primary human hematopoietic stem cells and progenitor cells (HSPCs) as the cell source of CAR-M. CAR-M derived from cord blood hematopoietic stem and progenitor cells (CB-HSPCs) demonstrated standard macrophage phenotypes, morphologies, and fundamental antimicrobial phagocytic capabilities in proof-of-concept experiments.

#### 3.2.3 Nanobiomaterials gene transfer vector

**FIGURE 8:**
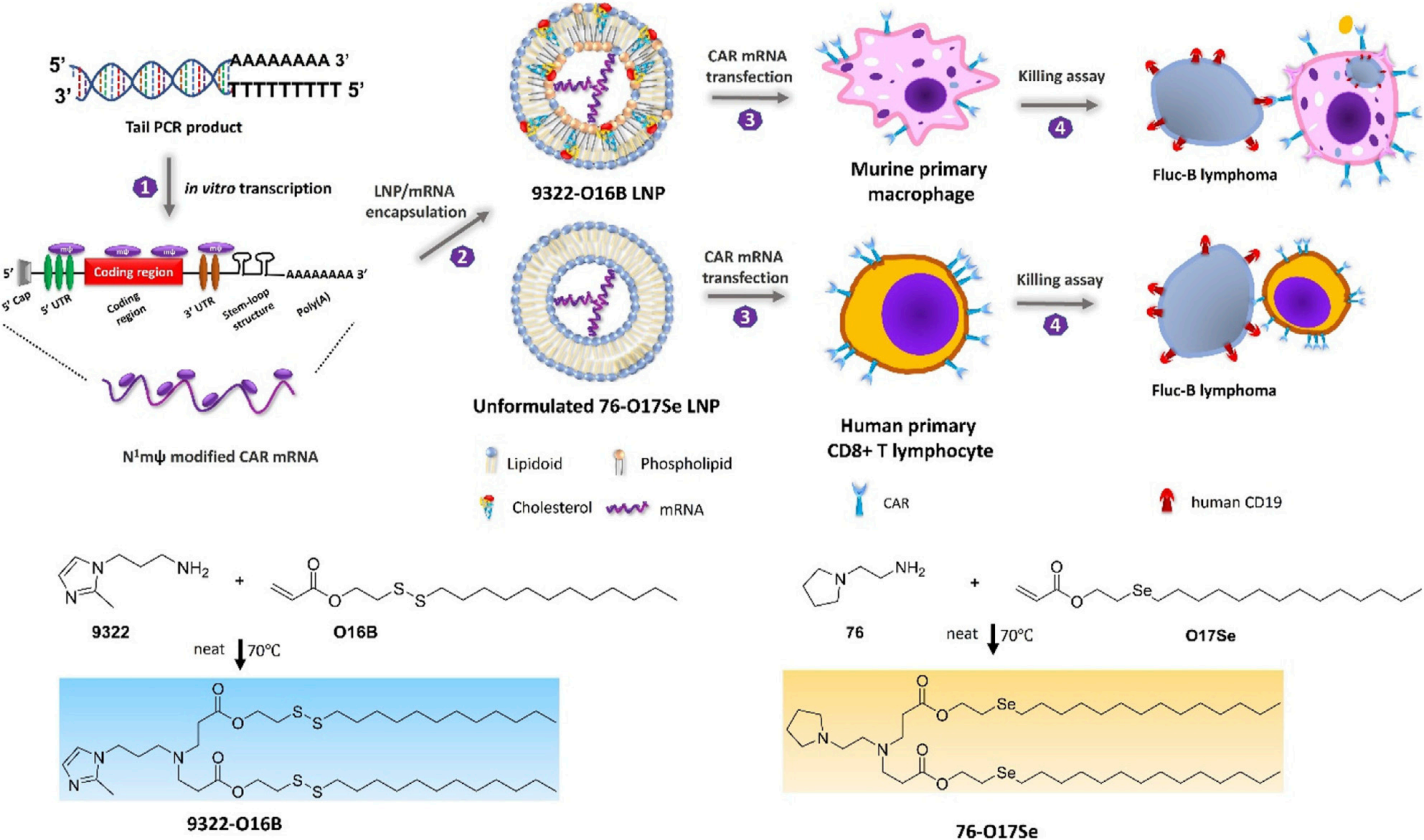
Schematic depicting N1mψ-modified CAR mRNA delivery to murine primary macrophages and human CD8 + T-cells (Ye et al., 2022). Copyright^©^ 2022, ACS.

**FIGURE 9:**
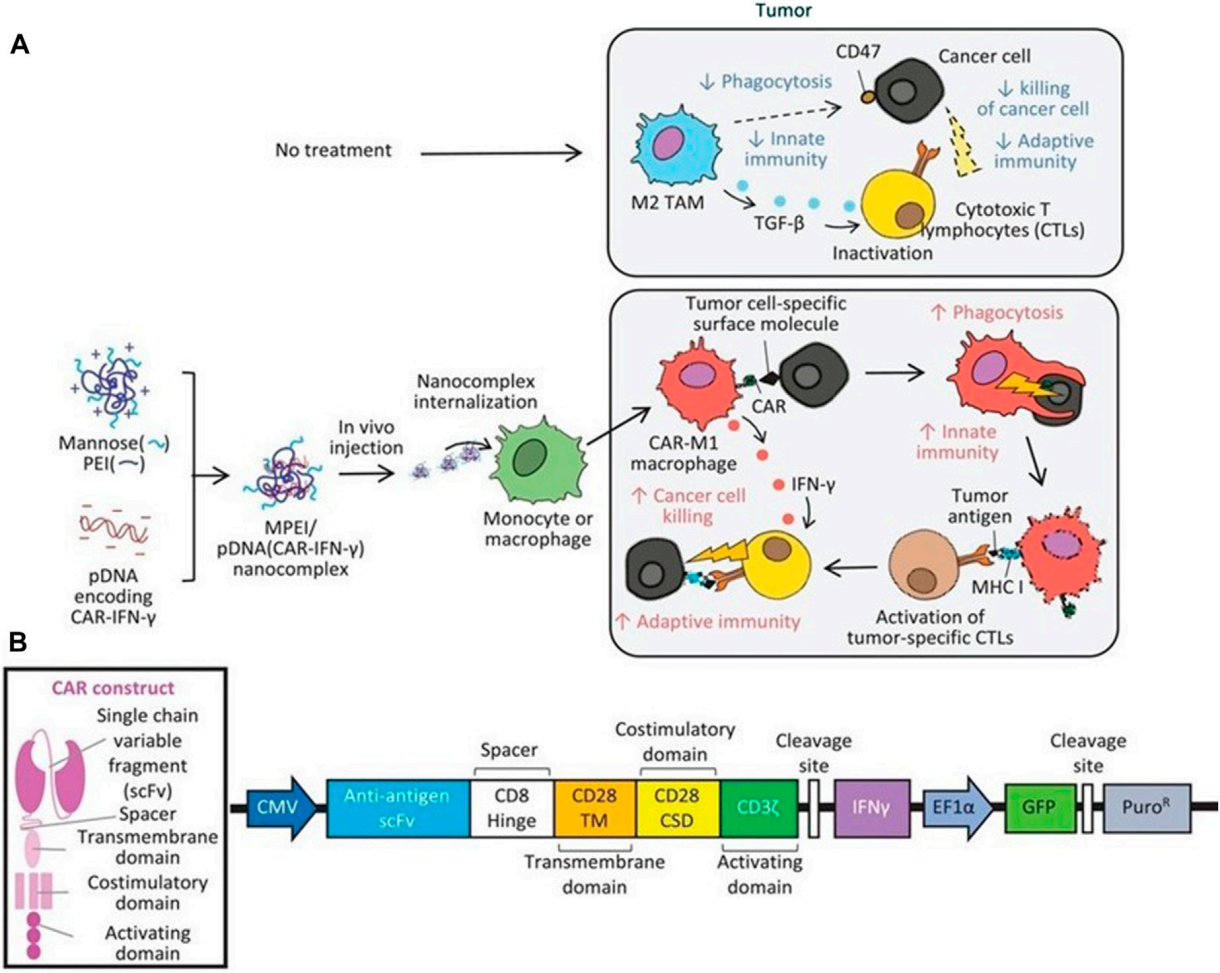
Therapeutic mechanisms of the MPEI/pCAR-IFN-γ nanocomplex. **(A)** Schematic illustrating the delivery of combinatorial gene encoding ALK-specific CAR and IFN-γ plasmid DNA (pCAR-IFN-γ) using MPEI to induce CAR-M1 macrophages *in vivo*, as well as, the anti-tumoral mechanisms of CAR-M1 macrophages. **(B)** Transgene construct of the combinatorial gene expressing anti-ALK CAR and IFN-γ (Kang et al., 2021). Copyright^©^ 2021, Wiley.

**FIGURE 10:**
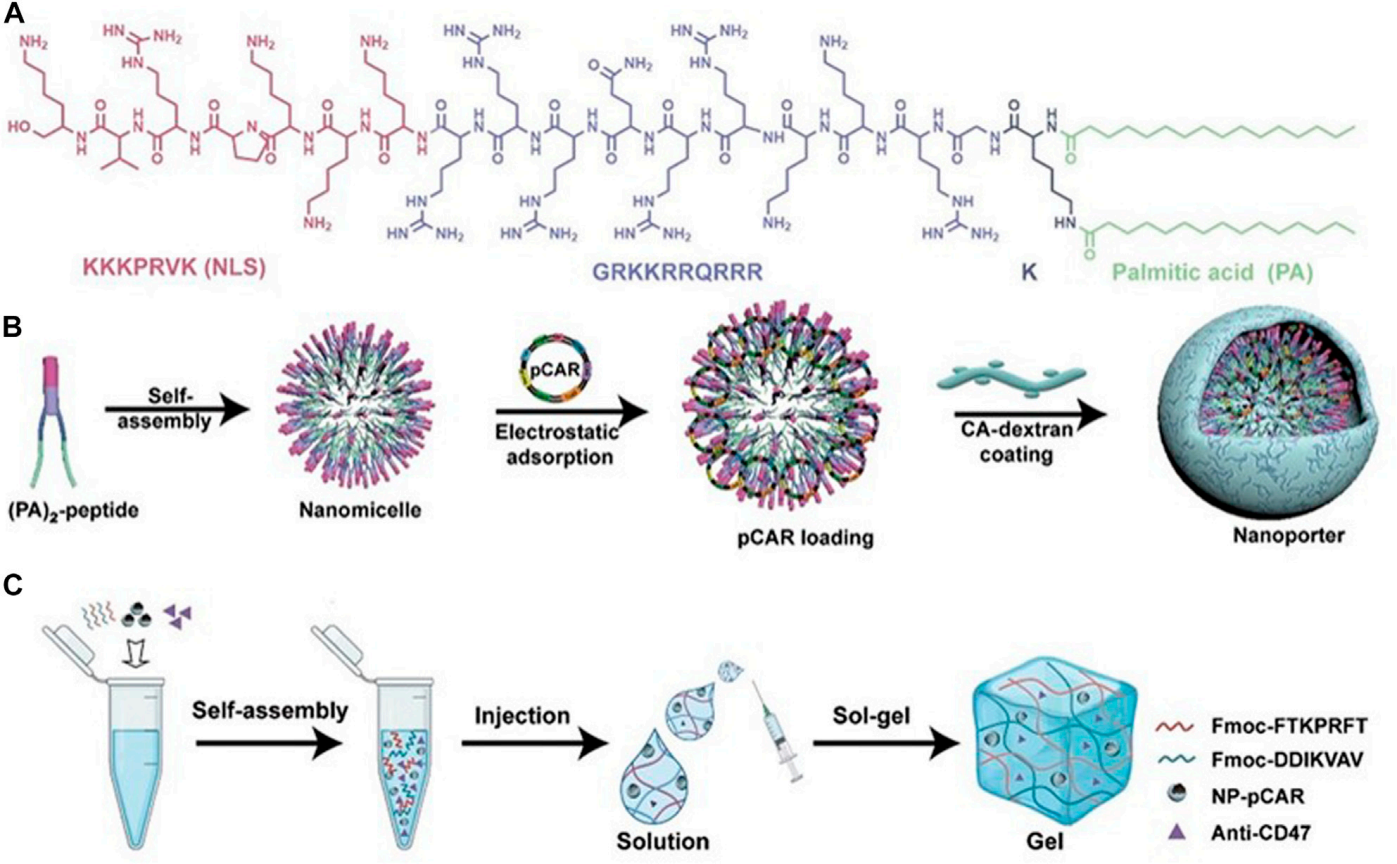
Design schematic of the cavity-injectable superstructure. **(A)** The chemical structure of (PA)_2_-peptide. **(B)** Schematic illustrating the preparation of pCAR-laden nanoporters (NP-pCAR). **(C)** Schematic illustrating hydrogel for the encapsulation of NP-pCAR and anti-CD47 (Chen et al., 2022a). Copyright^©^ 2022, AAAS.

In consideration of whether CAR-M could be successfully transferred to the clinic, the production cost and application safety also need to be careful consideration. All of the above-mentioned attempts for CAR-M are the use of viral vectors to genetically engineer macrophages, just like the CAR-T products currently on the market. It is worth noting that the utilization of viral vectors such as lentivirus or adenovirus to carry CAR gene sequences can indeed efficiently transduce macrophages and enable them to efficiently express chimeric antigen molecules to perform functions, but at the same time, the high cost and safety problems introduced will greatly limit the development of CAR-cell technology and its widespread application in patients. Hence, exploring a new vector with a simple synthesis process, economic cost, and high gene delivery efficiency has emerged as a crucial research direction in the advancement of CAR-M technology. Under this demand, nanobiomaterials have shown unique advantages, and a number of non-viral vectors represented by nanobiomaterials have been applied in CAR-cell therapy. Among them, lipid nanoparticle (LNP) vectors loaded with mRNA vaccines have shined in the vaccine research and development of the new crown epidemic, and more and more researchers have begun to pay attention to the development and application of LNP vector technology (Rurik et al., 2022; Ye et al., 2022), including the application of LNP-mRNA technology in CAR-T and CAR-M. For instance, Ye et al. (2022) successfully engineered CAR-M and CAR-T-cells using LNP-loaded CAR mRNAs (Figure 8). In this study, they performed mRNA transfection of T-cells and macrophages using an optimized LNP formulation, and the resulting CAR-T and CAR-M cells showed a significant cytotoxic effect on B lymphoma *in vitro*, highlighting the great potential of LNP-mRNA technology for applications to engineered adoptive cell therapies. In another interesting study (Zhou et al., 2022), zhou and colleagues constructed an LNP system with CD3 antibody modification for targeted transfection to generate CAR T-cells. Using the system to deliver a combinatorial gene of interleukin 6 short hairpin RNA (IL-6 shRNA) and CD19 CAR, they successfully transformed T-cells into IL-6 downregulated CAR-T-cells, which killed leukemic tumor cells with high CD19 expression while reducing the CRS caused by IL-6. However, both these CAR-M and CAR-T cells-based production strategies of using LNPs still took the old path: first acquiring T-cells or macrophages, then introducing tumor antigen-specific CAR genes *in vitro*, and reinfusing CAR-T/CAR-M back into the patient’s body after amplification. The course of treatment took a long time and required cellular modification in facilities that meet specific production quality management specifications (GMP), which was still very expensive. To address these problems, CAR-M therapies may also be attempted towards the ready-to-use and towards the direct generation of CAR-M *in vivo*. The novel concept of the acquisition of CAR-M *in vivo* through non-viral vectors has been used in developing a new generation of CAR-M technology, hoping to combat the above problems through a cost-effective and simple manufacturing process. Kang et al. (2021) attempted to use macrophage-targeted polymer nanocarriers (MPEI/pCAR-IFN-γ) to introduce the combinatorial gene encoding CAR and IFN-γ into macrophages *in situ* to avoid the complex and expensive *in vitro* preparation process (Figure 9). Tumor-bearing mice were implanted with macrophage-targeting nanocarriers and CAR-encoded plasmid DNA nanocomposites, then CAR-M1 macrophages were induced to form *in vivo* and mediated tumor phagocytosis, anti-tumor immunomodulation, and restrained solid tumor growth. With further assistance with cytokines, this strategy improved the immunomodulatory and tumoricidal capacity of CAR-M products. This study innovatively demonstrated the feasibility of the acquisition of CAR-M *in vivo* through non-viral nanobiomaterials vectors. At the same time, some researchers were trying other immunotherapy therapies based on CAR-M technology *in vivo*. Chen et al. (2022a) developed an injectable hydrogel “drug reservoir” system that co-delivers macrophage-targeted edited nanocarriers (pCAR-NPs) and CD47 antibodies in a “filled form” in the postoperative tumor cavity of GBM (Figure 10). The pCAR-NPs perform *in situ* edited on the “local” macrophage around the postoperative tumor cavity, generating CAR-M around the tumor cavity that targeted the clearance of glioma stem cells (GSCs), meanwhile, CD47 antibodies block the “do not eat me” signal in tumors, thereby synergistically augmenting the phagocytic efficiency of CAR-M against GSCs and activating the adaptive immune system by using its antigen presentation effect simultaneously. These synergistic responses triggered the immune memory effect post-treatment and avoided the recurrence of gliomas. Therefore, the nanobiomaterials vector *in vivo* transfection strategy could potentially overcome the high costs and exhaustive process involved in traditional CAR-cell manufacturing, while circumventing the safety concerns induced by viral vectors *in vivo* representing. The novel gene transfer vectors provided an ideal alternative and powerful genetic modification tool for future CAR-M therapy strategies to conquer solid tumors.

## 4 Clinical attempts of CAR-Macrophages

Clinical translations of CAR-M therapies are still in the initial exploratory stages (Table 1), and until now, only two clinical trials have been disclosed on CAR-M therapy (NCT05007379 and NCT04660929), but yet still lack therapeutic outcomes. For instance, MaxCyte has developed an MCY-M11 that utilizes mRNA-transfected peripheral blood monocytes (PBMCs), the precursors of macrophages, for the production of CAR-M cells that target mesothelin-expressing cancer cells. This CAR therapy provides transient expression without the necessity of viral vectors or cell amplification via the utilization of MaxCyte GT System™, a cGMP-compliant electroporation system. This system enables rapid manufacturing and timely administration to the patient (Hung et al., 2018). Currently, a phase I clinical trial (NCT03608618) is underway to evaluate the efficacy of this treatment for advanced ovarian cancer and peritoneal mesothelioma. The drug candidate CT-0508, from CARISMA Therapeutics, is designed for the treatment of patients with relapsed/refractory HER2 overexpression (NCT04660929). This study recruited 18 patients with HER2-positive solid tumors for the first time to investigate the effects of CAR-M transduced by adenovirus. Recently, Carisma Therapeutics presented data from the phase I clinical trial of CT-0508 in patients with HER2-positive solid tumors at the 2023 American Society of Gene and Cell Therapy (ASGCT). The results highlighted the preliminary outcomes of CT-0508 in 18 patients with locally advanced (unresectable) or metastatic HER2 overexpressing solid tumors, focusing on aspects such as safety, tolerability, feasibility of cell manufacturing, transportation, and activation of the TME. However, in this clinical trial, patients need to be injected with hundreds of millions of engineered macrophages at a time, and considering the difficulty of *in vitro* expansion of macrophages discussed earlier, the application prospects of CT-0508 are undoubtedly challenging. It is worth noting that several companies have designed CAR-M pipelines in advance according to different molecular targets, cell sources, and transduction vectors. These ongoing results present the possibility of the advancement of CAR-M immunotherapeutic modalities for the treatment of solid tumors.

**TABLE 1:** Summary of CAR-M-based preclinical studies and clinical trials.

Target antigen	Intracellular domain	Gene delivery	Macrophage source	Application strategy	Clinical trials	References
CEA	CD64	adenoviral vector	Monocytes derived from human peripheral blood mononuclear cells (PBMCs)	*in vitro*	-	Biglari et al. (2006)
CD19 CD22	Fc Gamma	lentiviral vector	J774A.1 Macrophages Bone marrow derived macrophages (BMDMs)	*in vitro*	-	Morrissey et al. (2018)
	Megf10					
	MerTK					
	Bai1					
	CD3zeta					
	PI3K					
HER2	CD147	lentiviral vector	Raw264.7	*in vitro*	-	Zhang et al. (2019)
CCR7	TLR2	lentiviral vector	RAW264.7	*in vitro*	-	Niu et al. (2021)
	TLR4					
	TLR6					
	MerTK					
	4-1BB-CD3ζ					
CD19 ALK	CD28^−^CD3ζ	Non-viral vector: jetPEI-macrophage (MPEI)	RAW264.7	*in vivo*	-	Kang et al. (2021)
			M2 BMDM			
			M2 TAMs			
CD133	CD3ζ	Non-viral vector: nanoporter (NP)-hydrogel	THP-1 bone marrow-derived macrophages (BMDMs) RAW 264.7	*in vivo*	-	Chen et al. (2022a)
CD19	CD3ζ	Non-viral vector: Lipid nanoparticle (LNP)	RAW264.7 bone marrow-derived macrophages (BMDMs)	*in vitro*	-	Ye et al. (2022)
Mesothelin	CD3ζ	MaxCyte GT System™	Peripheral blood monocytes	*in vitro*	NCT03608618	Hung et al. (2018)
CD19	CD3ζ	adenoviral vector (Ad5f35)	THP-1 monocyte-derived macrophages	*in vitro*	NCT04660929	Klichinsky et al. (2020)_
Mesothelin						
HER2						
HER2	CD3ζ	adenoviral vector (Ad5f35)	Peripheral blood monocytes	*in vitro*	-	Gabitova et al. (2021)
CD19 meso	CD86-FcγR I	lentiviral vector	iPSCs from peripheral blood mononuclear cells (PBMC)	*in vitro*	-	Zhang et al. (2020)
CEA	CD28^−^CD3ζ	lentiviral vector	THP-1 hematopoietic stem and progenitor cells (HSPCs)	*in vitro*	-	Paasch et al. (2022)

## 5 Summary and outlook

As an essential function in the innate immune system and adaptive immune system, macrophages have shown great potential for synergistic therapy in solid tumors. The emerging cellular immunotherapy approach, CAR-M, provides a novel and promising solution in cancer immunotherapy. Importantly, the nanobiomaterials-based gene transfer vector solved the technical barriers to achieving CAR-M therapy *in situ*, in addition to cost and safety. Although this paradigm has been validated in several tumor-bearing mice models, many challenges still need to be overcome before clinical applications. First, the efficiency of gene reprogramming determines the therapeutic effect of CAR-M, so the nanobiomaterials maybe need to provide efficient target cell transfection. Taking *in vitro* viral infection as an example, compared with T-cell infection, the use of viral vectors to infect macrophages is extremely inefficient, which means that a larger dose of virus is required to achieve the expected therapeutic effect, which greatly increases the cost of treatment. Second, the pharmacokinetics behavior of biomaterials gene vector *in vivo* is also worth considering, especially *in vivo* CAR-M protocol, after systemic administration, most of the exogenous vehicles will be distributed in the liver, failing to reach the tumor lesion and affecting the treatment effect. Third, solid tumor-specific targets determine the safety of CAR-cell immunotherapy *in vivo*. Just as in CAR-T therapy, if the specificity of the selected target is not high enough, then it may bring very serious toxic side effects, and the tumor cells with lower target antigen expression may also evade the pursuit of CAR-M and cause tumor recurrence. Therefore, the rational design of delivery vehicles for efficient targeting of special cells poses a great challenge in this field again. Last, the complex microenvironment of solid tumors may impair the therapeutic efficacy of CAR-M. Although macrophages have a stronger tumor infiltration capacity than T-cells, and CAR-M has shown promising results in mice tumor models in preclinical studies, considering that human tumor microenvironments are much more complex than those of animal models, so how to improve the infiltration of CAR-M in tumors will also be a problem to achieve therapeutic effects. The *in situ* reprogramming of macrophages avoids complex isolation and reprogramming operations *in vitro* to a certain extent, is not affected by the migration distribution of CAR-M, and has a certain degree of advanced application, but still faces the problem of *in vivo* delivery of reprogrammed genes. For a virus-depend delivery protocol to be adopted, the viral vector must have excellent *in vivo* safety and be able to accurately target macrophages to reprogram them when entering the complex physiological environment. For non-viral vector delivery, under the premise of solving the same challenges as viral vectors, the transfection efficiency and the potential cross-reaction between target cells and tumor microenvironment triggered by nanobiomaterials remain must be improved to meet the requirement of treatment.

After solving the above-mentioned challenges, the optimized CAR-M technology platform is expected to truly become a “sharp blade” for solid tumor treatment. Significantly, the development of the future CAR-M technology platform cannot simply follow the old route of CAR-T technology iteration. The structure of the CAR is originally designed to activate the function of T-cells, theoretically, the novel and efficient CAR molecules should be explored to activate the function of macrophages. Because the function of macrophages in maintaining homeostasis is more complex than that of T-cells and NK cells, the future application of the CAR-M technology platform may not be limited to a simple cell or reprogramming factor infusion. These special functions provide a new focus for applying a new generation of CAR-M technology. In addition, CAR-M has great potential in modifying the solid TME, which provides the possibility of combining CAR-M therapy with other therapies. Recently, benefiting from the rapid development of nanotechnology in cancer theranostics, the possibility of using nanobiomaterials vehicles to achieve CAR-M *in situ* in an efficient, economical, and specific manner against solid tumors is desired.
